# Comparison of capillary and venous hemoglobin determination among school-age children: insights from Zanzibar, Tanzania

**DOI:** 10.1515/almed-2025-0053

**Published:** 2026-04-15

**Authors:** Kaunara Azizi, Tedson Lukindo, Stanslaus Hendry, Asha Salmin, Francis Millinga, Erick Killel, Kombo Mdachi, Hawa Msola, Ray Masumo, Ramadhani Mwiru, Ramadhani Noor, Patrick Codjia, Germana Leyna, Geofrey Mchau

**Affiliations:** Department of Food Science and Nutrition, 127806 Tanzania Food and Nutrition Centre, Dar es Salaam, Tanzania; Department of Community Health and Nutrition, 127806 Tanzania Food and Nutrition Centre, Dar es Salaam, Tanzania; Nutirition Unit, Ministry of Health, Zanzibar, Tanzania; Office of the Chief Government Statistician, Zanzibar, Tanzania; United Nations Children’s Fund (UNICEF), Zanzibar, Tanzania; Department of Statistics, University of Dar es Salaam (UDSM), Dar es Salaam, Tanzania; United Nations Children’s Fund (UNICEF), Dar es Salaam, Tanzania; Department of Epidemiology and Biostatistics, Muhimbili University of Health and Allied Sciences, Dar es Salaam, Tanzania

**Keywords:** anemia, capillary blood, hemoglobin, venous blood, Tanzania

## Abstract

**Objectives:**

There is apprehension regarding the agreement between capillary and venous hemoglobin (Hb) concentrations, which may have far-reaching consequences for global anemia prevalence estimates, heavily dependent on capillary Hb data.

**Methods:**

We investigated the discrepancies in Hb concentrations obtained from venous and capillary blood samples collected among school-age children in Zanzibar. Hb concentrations were measured in 810 children aged 10–19 years old. Both capillary and venous Hb measurements used a HemoCue 201+ analyzer (HemoCue, Angelholm, Sweden). Hb means, standard deviations, and anemia prevalence were analyzed using Stata. The means of the anemia categories were compared using paired t-tests. The agreement between capillary and venous Hb measurements was assessed using Pearson’s correlation coefficients, a Passing-Bablok regression, and a Bland-Altman plot.

**Results:**

The correlation between paired measurements of capillary and venous Hb concentration was high, r=0.72 (p<0.001). The prevalence of anemia using venous samples was 20.5 %, and using capillary samples was 44.7 %. Mean Hb concentrations were higher in venous than capillary samples (mean ± SD: 12.994 ± 1.54 vs. 12.085 ± 1.36 g/dL, p<0.001). The mean difference between the methods is −0.909 g/dL, and the limits of agreement are (−3.065 – 1.247). Passing-Bablok regression analysis showed no evidence of systematic error between capillary and venous hemoglobin measurements.

**Conclusions:**

Capillary and venous hemoglobin measurements differ significantly; capillary Hb cannot replace venous Hb for accurate anemia assessment.

## Introduction

Anemia is a pervasive public health issue that affects women and children, particularly school-age children in developing countries [1], [2], [3]. The World Health Organization (WHO) report estimates that globally, 25.4 % of school-age children (SAC), and 47.4 % of preschool children under 5 years old are affected by anemia [2]. In Tanzania, Demographic and Health Surveys report a prevalence of 59 % among under-five children [4]. Findings from the 2019 Tanzania School Malaria and Nutrition Survey, and a cross-sectional survey by Yusufu et al. in Zanzibar revealed that 33.7 % and 53.3 % of school-aged children and adolescents are anemic [5], 6]. Anemia during early life can have serious repercussions later in life, including increased susceptibility to infections and chronic diseases, delayed cognitive and physical development, and decreased economic productivity at both the individual and societal levels [7], 8]. Additionally, chronic anemia may impede physical growth during all stages of development [7].

Hemoglobin (Hb) concentration is often used to diagnose anemia and assess its severity [9]. Venous blood samples obtained with automated hematology analyzers are considered the gold standard for anemia diagnosis [10], 11]. However, most widespread protocols for assessing Hb in field settings utilize the portable photometer, HemoCue Hb analyzer, which involves obtaining capillary blood via finger-prick or venous blood, and assessing the sample on a portable point-of-care photometer [12], [13], [14]. The portable Hb photometer system has become increasingly popular in recent years due to its portability, low cost, ease of use, lack of need for refrigeration or electricity, and immediate digital display of results [15], 16]. The diagnosis of anemia based on Hb concentration in venous or capillary blood is used to measure the prevalence of anemia and direct resources to affected groups. It is also used to screen individuals for program participation and evaluate responses to interventions [17]. Precise estimations of the prevalence and distribution of anemia in populations are necessary for the design of programs and interventions [10].

Blood samples obtained from capillaries or veins are analyzed to ascertain the hemoglobin concentration, but the results obtained are often not correlated to the sample source [18]. Venous blood sampling requires specialized materials and trained personnel, and can cause post-venipuncture complications; finger-stick sampling is a simpler procedure, but has a limited blood volume which is traditionally insufficient for analysis [19]. Studies comparing hemoglobin concentration in capillary and venous blood collected at the same time in the same population indicate that the difference between these two blood sources vary. In some cases, venous blood has higher hemoglobin concentration, while in other, capillary blood shows higher hemoglobin concentration [10], [19], [20], [21]. This variability can impact estimate of anemia prevalence depending on the blood type used, this might even change how serious anemia is considered for public health in a country.

The disparity between capillary and venous blood hemoglobin estimations has been reported in numerous studies [10], 17], 20], 22], 23]. However, there has been no available information regarding this discrepancy among school-age children in Zanzibar, Tanzania. Therefore, this study aims to investigate the extent of this disparity by comparing the Hb concentrations of capillary vs. venous blood samples obtained from school-age children in Zanzibar, Tanzania, using the HemoCue Hb 201+ analyzer.

## Materials and methods

### Subject and study setting

A school-based cross-sectional study was conducted from January to March 2022 in Pemba and Unguja, two islands located off the East African coast, in the Indian Ocean. They are part of the Zanzibar Archipelago, which is situated in Tanzania, approximately 25 miles from the mainland. Pemba and Unguja have a tropical climate, characterized by hot, humid, and wet weather. Both islands are home to various flora and fauna, including coral reefs and tropical forests. The islands are also known for their unique cultures and traditions, which reflect the diverse ethnic groups that inhabit the area. This study was conducted in 70 selected primary and secondary schools. Subjects were school-age children (5–19 years old). A sub-sample of 810 students aged 10–19 years were selected for this present study. Participants’ sociodemographic characteristics, including age, sex, education, and residence, were sought by trained research assistants using semi-structured questionnaires.

### Sample collection and laboratory analysis

Blood samples were collected from consented participants at the school after they were informed of the study procedures. For every participant, four mL of venous samples were collected directly into purple vacutainer tubes (Becton Dickinson, New Jersey, USA) containing spray-dried dipotassium EDTA (K_2_EDTA) at a concentration of 1.8 mg/mL of whole blood. The vacutainer tube was inverted to ensure an adequate mixture with the anticoagulant. The tubes were maintained at 4–8 °C for less than 2 h before shifting to the laboratory for further processing. At the laboratory, venous blood samples were processed to estimate their Hb concentration using the HemoCue HB 201+ analyzer (HemoCue, Angelholm, Sweden).

A capillary blood sample was obtained via finger-pricking following disinfection of the fingertip with a swab contains 70 % isopropanol-alcohol (Heinz Herenz Hamburg, Germany). The first drop of blood was removed with a sterile cotton swab, and the remaining blood was collected directly from the finger by capillary action into the HemoCue microcuvettes. The microcuvettes are designed to draw blood using capillary action, allowing a small volume of blood (∼10 µL) to be drawn into the microcuvette. Subsequently, Hb concentration was determined photometrically using the HemoCue Hb 201+ analyzer (HemoCue, Angelholm, Sweden). Care was taken throughout the procedure to avoid any pressure on the finger to prevent hemodilution due to the inclusion of interstitial and intracellular fluids.

School-age children with Hb concentration lower than 12 g/dL were considered as anemic. Mild anemia was defined as an Hb concentration of 11–11.9 g/dL. Moderate anemia was defined as Hb concentration between 8 and 10.9 g/dL, and severe anemia was defined as Hb concentration lower than 8 g/dL [9]. Our classification of anemia was based on WHO 2011 thresholds, as the analysis was completed prior to the release of the updated WHO guideline (2024). The new recommendations provide age- and sex-specific cut-offs that may yield different prevalence estimates [24].

### Quality control

Imprecision was assessed using daily control from the HemoTrol quality control solutions, which consist of three levels of hemoglobin concentration (low, medium, and high). These control samples were analyzed to monitor measurement consistency and assess variability. The imprecision was calculated based on repeated measurements performed over multiple days, allowing for the evaluation of both within-day and between-day variability. The coefficient of variation (CV) for each control level was determined to quantify analytical precision, ensuring reliability in the comparison between capillary and venous hemoglobin determination.

The accuracy of the HemoCue 201+ analyzers was evaluated prior to the study by comparing results against a Sysmex XP-300 hematology analyzer at the Tanzania Food and Nutrition Centre Laboratory.

### Statistical analysis

Data were cleaned and analyzed using Stata version 17(Stata Corp LLC, USA). Descriptive statistics (mean and standard deviation) were computed for continuous variables. Pearson’s correlation coefficients, a Passing-Bablok regression, and a Bland-Altman plot were used to assess agreement between capillary and venous Hb measurements. p-values, and confidence intervals were used to show associations between variables. A p-value of <0.05 was considered statistically significant.

## Results

Hemoglobin concentration from capillary (finger stick) and venous samples were available for a total of 810 adolescents. The mean age for adolescents was 13.74 ± 2.396 years. The mean Hb concentration was 12.085 ± 1.36 g/dL and 12.994 ± 1.54 g/dL for capillary and venous samples, respectively (Table 1).

**Table 1:** Descriptive characteristics of study participants.

Characteristics (n=810)	Mean	SD (range)
Age, years, mean ± SD (range)	13.742	2.396 (10–19)
Capillary, g/dL, mean ± SD (range) Venous, g/dL, mean ± SD (range)	12.085 12.994	1.363 (7.2–17.4) 1.537 (6.9–18.6)

**Figure 1:**
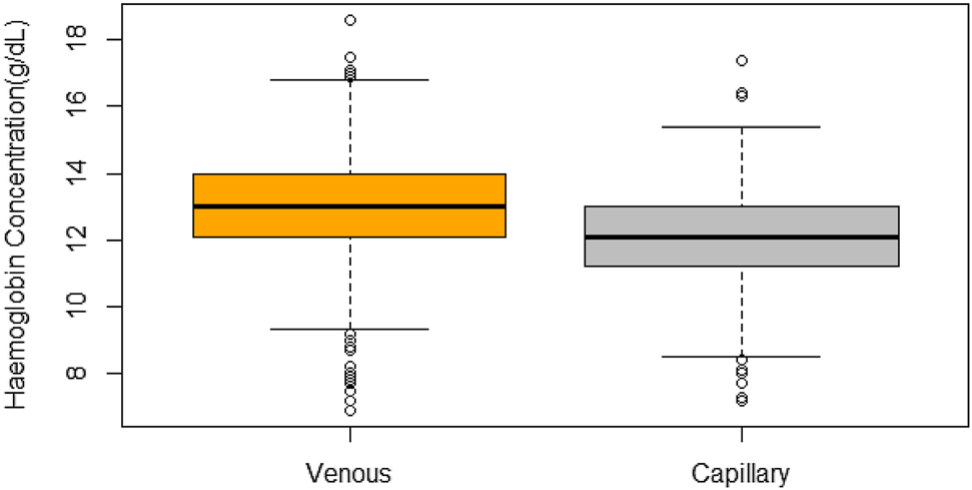
Distribution of hemoglobin measurement obtained by capillary and venous HemoCue.

Figure 1 illustrates the dispersion of values for both measurement methods, higher values of Hb concentrations can be observed in venous measurements. The bootstrap method was used to compare the CV for capillary sample readings (0.1128, 95 % CI: 0.1062–0.1194) and venous sample readings (0.1183, 95 % CI: 0.1107–0.1259). Although venous sample readings showed slightly higher relative variability, the difference in CVs (−0.0055, 95 % CI: −0.0116 to 0.0007) was not statistically significant (p=0.083) (Table 2).

**Table 2:** Mean Hb concentration (g/dL) across independent variables.

	Capillary blood n=810	95 CI	Venous blood n=810	95 CI	p-Value
Hb mean	12.085	11.99–12.18	12.994	12.89–13.10	0.001^a^
Hb range	7.2–17.4		6.9–18.6		
Anemia, %	362 (44.69)	41.27–48.12	166 (20.49)	17.71–23.27	0.001^b^
Mild	212 (26.17)	23.15–29.20	100 (12.35)	10.08–14.61	0.001
Moderate	146 (18.02)	15.38–20.67	60 (7.41)	5.60–9.21	0.001
Severe	4 (0.49)	0.01–0.98	6 (0.74)	0.15–1.33	0.5258

**Correlation**					

Estimate, r	0.72		0.72		0.001
CV, %	11.28		11.83		0.083

^a^t-Test to check the difference in means between the two variables.^b^Proportions test to check associations within variable groups.

**Figure 2:**
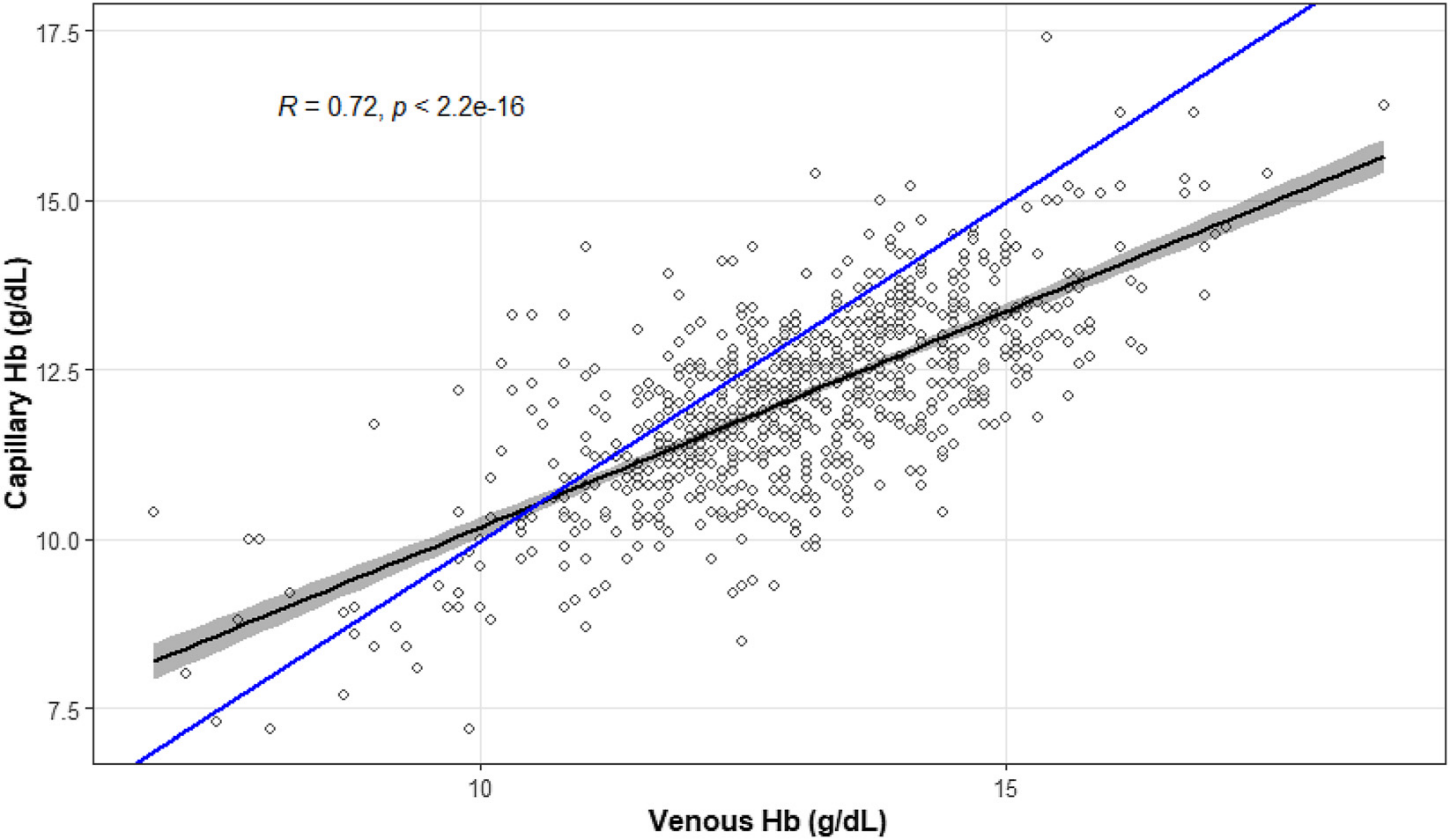
Correlation plot between venous and capillary Hb estimation.

The correlation between paired measurements of capillary and venous Hb concentration was high, r=0.72 (p<0.0001). A scatter plot to compare the two methods is presented in Figure 2.

The prevalence of anemia was found to be 44.69 % using capillary measurement and 20.49 % in venous Hb measurement. There was a significant difference in the proportion of anemic adolescents between capillary and venous blood samples (p<0.0001). A significant difference was observed in the proportion of adolescents classified with mild anemia, 26.17 % [95 CI, 23.15–29.20] for the capillary sample compared to 12.35 % [95 CI, 10.08–14.61] in the venous sample. Similarly, a higher proportion of adolescents were moderately anemic 18.02 [95 CI, 15.38–20.67] in capillary as compared to venous samples with 7.41 [95 CI, 5.60–9.21]. However, there was no statistically significant difference in the proportional of adolescents classified with severe anemia between capillary and venous mean Hb concentration (p>0.05) (Table 2).

Mean Hb concentrations were significantly higher in venous than capillary samples for the full sample (mean±SD: 12.994 ± 1.54 vs. 12.085 ± 1.36 g/dL, p<0.001), no anemia and mild anemia categories. In contrast, mean Hb concentrations were significantly higher in capillary than venous sample for severe anemia category (mean±SD: 9.083 ± 1.25 vs. 7.500 ± 0.38 g/dL, p<0.05), using venous sample to define anemia (Table 3).

**Table 3:** Hemoglobin concentration (g/dL) in capillary and venous blood samples.^a^

	Capillary blood	Venous blood	Paired t-test
Full sample	12.085 ± 1.36	12.994 ± 1.54	0.0001
No anemia	12.413 ± 1.17	13.519 ± 1.13	0.0001
Mild anemia	11.310 ± 0.91	11.680 ± 0.47	0.0001
Moderate anemia	10.153 ± 1.45	10.098 ± 0.73	0.7315
Severe anemia	9.083 ± 1.25	7.500 ± 0.384	0.0300

Anemia categories defined using the venous sample. ^a^Values are mean ± SD.

**Figure 3:**
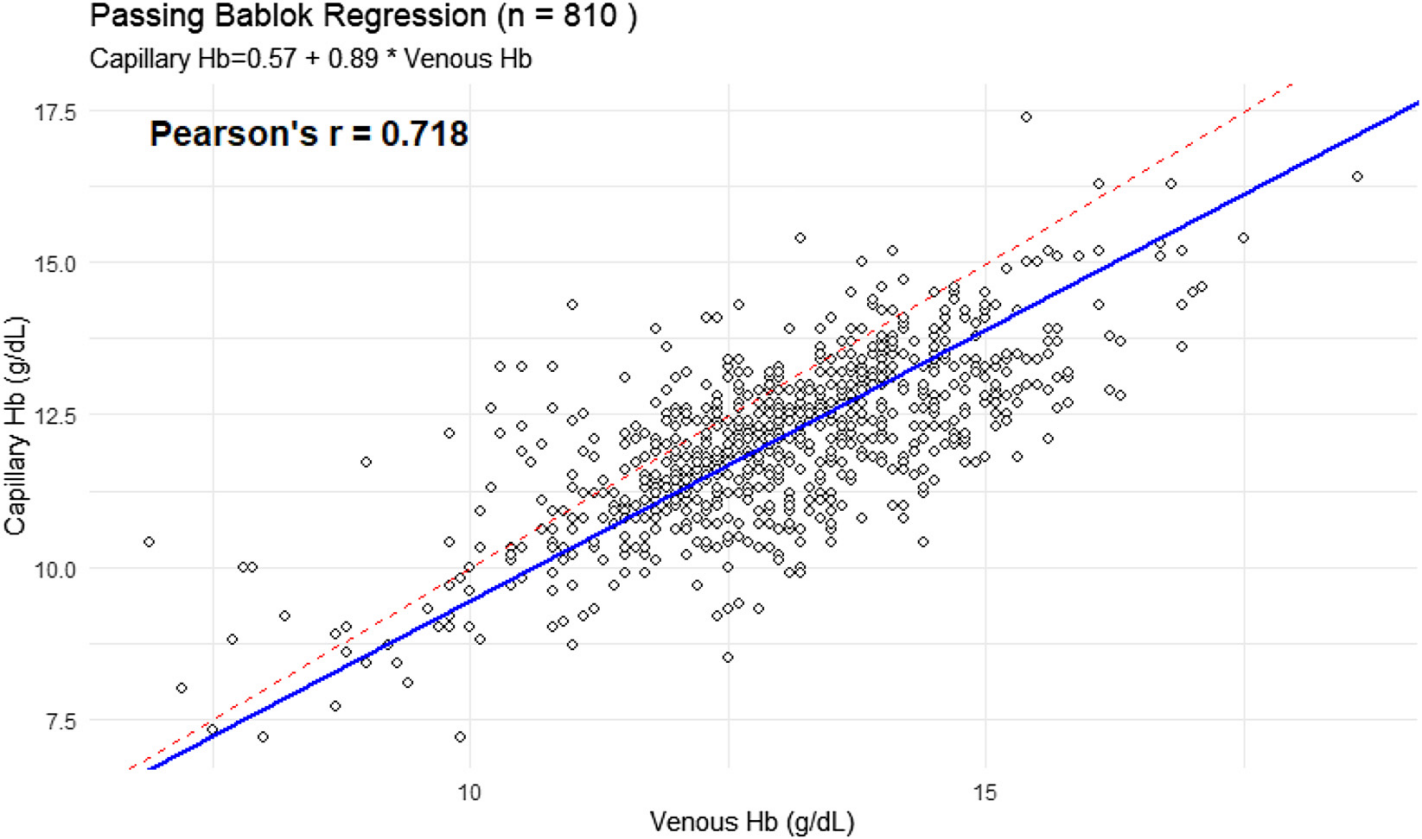
Passing-Bablok regression fit.

A Passing-Bablok regression was performed to compare measurements obtained from capillary and venous, using data from 810 paired observations. Passing-Bablok regression analysis showed no evidence of systematic error between capillary and venous hemoglobin measurements. The regression equation was Capillary Hb=0.57 + 0.89 × Venous Hb. The 95 % confidence interval (CI) for the intercept ranged from −0.17 to 1.20, which includes zero, indicating no significant constant bias between the two methods. In contrast, the 95 % CI for the slope (0.84–0.95) did not include 1.00, demonstrating the presence of a proportional difference, whereby capillary measurements increasingly underestimate venous hemoglobin as concentrations rise. The correlation between the paired measurements was moderate (Pearson’s r=0.718) (Figure 3).

**Figure 4:**
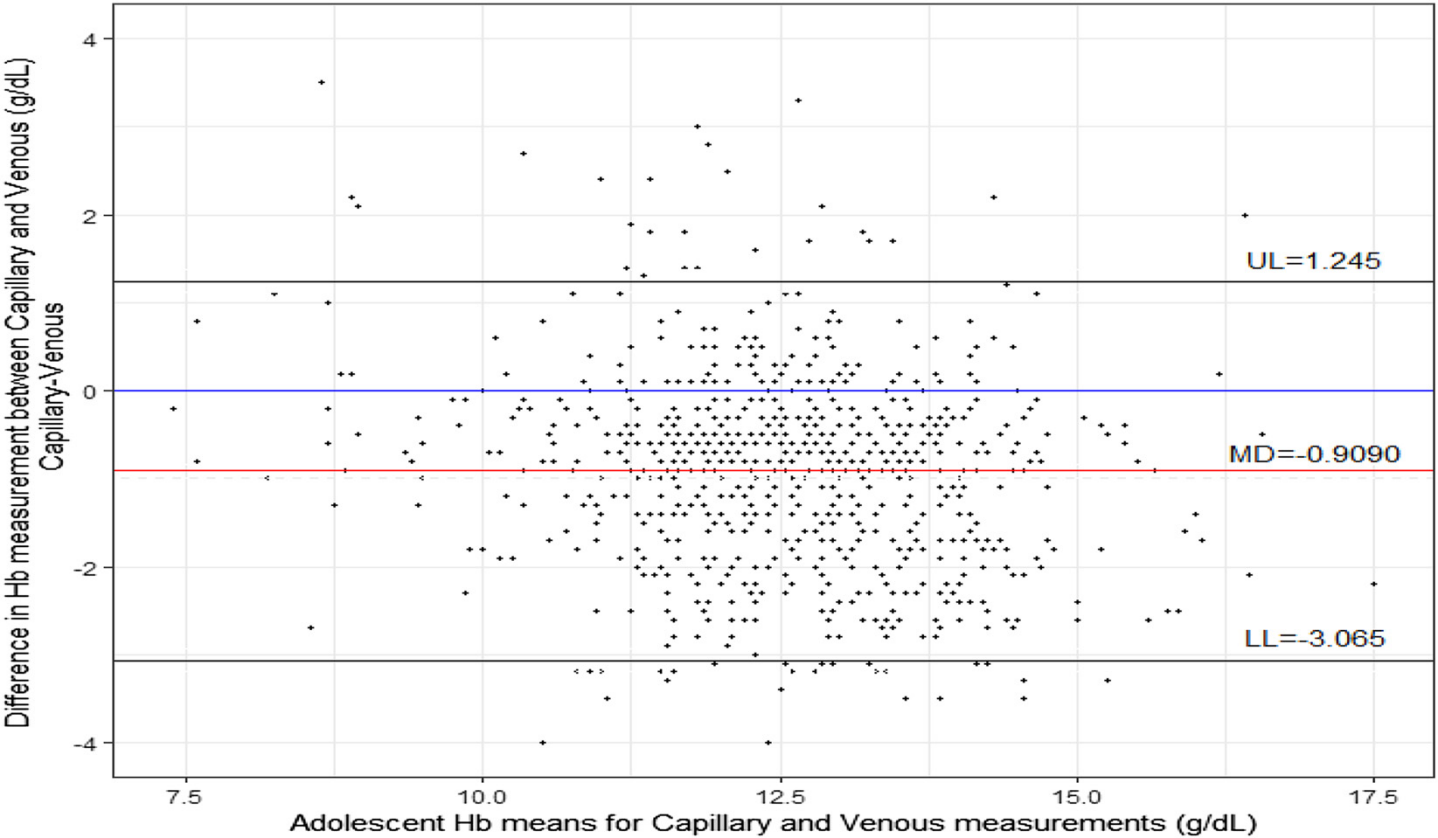
Bland-Altman plots for venous and capillary Hb bias measurements.

The Bland-Altman plot for comparison between capillary and venous Hb measurement is presented in Figure 4. The mean difference between the methods is −0.909 g/dL, and the limits of agreement is (−3.065-1.247).

## Discussion

Several studies have been reported on the disparity between capillary and venous blood hemoglobin estimations. In the current study, we report this discrepancy on hemoglobin estimation among adolescents aged 10–19 years in Zanzibar, Tanzania. To the best of our knowledge, this is the first study that compares capillary and venous blood hemoglobin estimation using HemoCue in Zanzibar, Tanzania. The study found that the estimated prevalence of anemia using capillary samples was 44.7 %, which was twice as high as the prevalence using venous samples (20.5 %). This underlines an alarming mismatch between the results obtained in Hb measurements depending on the type of sample employed. A minor variation in hemoglobin concentrations between the two sample types can lead to differing anemia classifications. According to WHO criteria [9], estimates based on venous samples suggest that anemia among school-age children in Zanzibar, Tanzania, represents a moderate public health concern. However, using capillary-based estimates, the classification would shift to indicate anemia as a severe public health issue.

The large disagreement in categorizing anemia status renders drawing conclusions from only the capillary method erroneous. This is consistent with previous studies that reported lower Hb concentrations in capillary samples compared to venous samples [10], 25]. The difference in Hb concentrations can be attributed to differences between the two sample types rather than the measurement methods. Capillary samples are taken from the fingertip, where the blood may have been diluted with tissue fluid, while venous samples are taken from the arm, where the blood has been oxygenated, resulting in a higher Hb concentration [26].

In this study, the mean concentrations of hemoglobin were compared between venous and capillary samples in different categories of anemia. The results show that for the full sample, including both anemic and non-anemic individuals, the mean Hb concentrations were significantly higher in venous samples compared to capillary samples. Furthermore, when looking specifically at the categories of anemia, it was found that the mean Hb concentrations were significantly higher in venous samples compared to capillary samples for the no anemia and mild anemia categories. In contrast, the mean Hb concentrations were significantly higher in capillary samples compared to venous samples for the severe anemia category. This may be due to the fact that in severe anemia, the Hb concentration is much lower, leading to a smaller margin of error in capillary samples as compared to venous samples.

The analysis of coefficients of variation for capillary and venous sample readings revealed comparable levels of variability, with venous samples exhibiting a slightly higher CV than capillary samples. Despite this difference, the observed variation between the two methods was small (−0.0055) and not statistically significant (p=0.083), suggesting that both sampling approaches are consistent and reliable in measuring Hb concentrations. Furthermore, the correlation between paired measurements of capillary and venous Hb concentration was found to be high, with a significant value of 0.72. This indicates a positive, moderately strong linear relationship between the two methods. This finding aligns with previous studies that have reported a high correlation between the two methods [27].

Passing-Bablok regression analysis demonstrated that capillary and venous hemoglobin measurements showed no evidence of constant systematic bias across the measurement range. The confidence interval for the intercept included zero, confirming the absence of a fixed offset between the two methods. This finding indicates that any discrepancy between capillary and venous sampling is not uniform across concentrations, thereby excluding the presence of a constant systematic error.

In contrast, the slope confidence interval excluded unity, providing clear evidence of a proportional measurement error. This proportional bias reflects a tendency for capillary hemoglobin values to underestimate venous concentrations progressively at higher hemoglobin levels. This concentration-dependent divergence carries important implications for contemporary laboratory practice, emphasizing how methodological choice and analytical interpretation can influence both clinical decision making and population-level assessments. The proportional error observed at higher hemoglobin concentrations is particularly consequential, as it increases the likelihood of misclassification when capillary sampling is used, especially near diagnostic cut-offs. Recognizing this pattern is essential for ensuring accurate anemia classification, safeguarding the reliability of measurements in both clinical and field settings, and supporting the validity of epidemiological reporting. These findings highlight the need for heightened methodological awareness when applying capillary hemoglobin assessments and reinforce the importance of selecting measurement approaches that align with the analytical demands of laboratory medicine.

The moderate correlation observed (r=0.718) further supports the need for cautious interchangeability between capillary and venous sampling methods, especially in population-based surveys or screening programs. This discrepancy suggests that capillary measurements require calibration or correction before they can be reliably substituted for venous sampling, particularly in cases where Hb thresholds influence clinical decision making. From a laboratory medicine perspective, the observed proportional bias necessitates methodological adjustment if capillary sampling is to be integrated into routine diagnostics. Although capillary hemoglobin assessment remains a practical tool for field-based anemia screening, these findings suggest that its diagnostic accuracy may be compromised.

The Bland-Altman plot was also used to compare capillary and venous hemoglobin measurements. The mean difference between the two methods was −0.909 g/dL, indicating that the capillary Hb measurements were on average lower than the venous measurements. The limits of agreement, which represent the range within which 95 % of the observations lie, were calculated to be (−3.065 to 1.247). This indicates that there is a considerable amount of variation between the two methods, with some measurements being as much as 3.065 g/dL lower or 1.247 g/dL higher when comparing capillary and venous Hb concentrations. Such discrepancies may have significant clinical implications, as even small differences in Hb concentrations can impact treatment decisions. Relying on capillary hemoglobin measurements can lead to misallocation of resources and suboptimal targeting of anemia prevention and control programs.

The use of venous blood hemoglobin as the gold standard for anemia diagnosis is imperative to ensure reliable and comparable data across studies and populations [23]. A paradigm shift towards venous hemoglobin measurement is vital to accurately assess the burden of anemia and inform effective interventions [17]. This transition requires robust capacity building, including training health workers on venous blood collection techniques and establishing adequate laboratory infrastructure. By adopting venous blood hemoglobin measurement as a standard practice in national surveys and epidemiological studies, Tanzania can make significant strides in accurately assessing the burden of anemia and implementing targeted interventions to address this pressing public health issue.

The notable strengths of our study encompassed a substantial sample size and the provision of significant findings regarding the use of venous and capillary samples for measuring Hb concentrations, particularly in the context of anemia. Despite these strengths, it is important to note that this study had some limitations. The study population consisted of individuals from a specific geographical region, which may limit the external validity of the findings. Furthermore, other factors such as age, gender, and health status of the participants were not taken into account, which could affect the results.

## Conclusions

Evidence from the current study suggests there are significant differences in Hb measurements between capillary and venous methods. The results support the notion that using venous samples provides a more reliable measurement of Hb concentrations. The use of capillary samples for categorizing anemia status can significantly overestimate anemia prevalence. There is a need to review laboratory procedures for anemia measurement to be able to have a true estimate of anemia burden and develop appropriate prevention and control actions to curb the prevailing anemia challenge.
